# Deep Infiltrating Endometriosis of the Left Ureter Managed with Laparoscopic Ureterolysis Combined with Allium Ureteral Self-Expandable Stent: A Case Report

**DOI:** 10.3390/jcm13226769

**Published:** 2024-11-11

**Authors:** Marcin Jozwik, Magdalena Miłobędzka, Joanna Wojtkiewicz, Jörg Neymeyer, Artur Jakimiuk, Maciej Jozwik

**Affiliations:** 1 Department of Gynecology and Obstetrics, Collegium Medicum, University of Warmia and Mazury in Olsztyn, 10-045 Olsztyn, Poland; 2 Scientific Circle of the Department of Gynecology and Obstetrics, Collegium Medicum, University of Warmia and Mazury in Olsztyn, 10-045 Olsztyn, Poland; magdamilobedzka@gmail.com; 3 Department of Human Physiology and Pathophysiology, School of Medicine, University of Warmia and Mazury, 10-082 Olsztyn, Poland; 4 Department of Urology, Charité-Universitätsmedizin Berlin, Corporate Member of Freie Universität Berlin, Humboldt-Universität zu Berlin, and Berlin Institute of Health, 10117 Berlin, Germany; 5 Department of Gynecology, Gynecological Oncology and Reproduction, National Medical Institute of the Ministry of Interior and Administration, 02-507 Warszawa, Poland; artur.jakimiuk@cskmswia.gov.pl; 6 Department of Gynecology and Gynecologic Oncology, Medical University of Białystok, 15-276 Białystok, Poland; maciej.jozwik@umb.edu.pl

**Keywords:** ureteral deep infiltrating endometriosis, hypertension, ureteral stent, ureteral stricture

## Abstract

**Introduction**: In endometriosis, urinary tract involvement occurs in 1–5.5% of cases, where the ureter is affected in 9–23%. Unfortunately, endometriosis may remain asymptomatic even with significant anatomical progression. A delay in the diagnosis and treatment of ureteral endometriosis may result in hydronephrotic kidney damage and functional impairment. **Methods**: We present a case of a 36-year-old woman with a left ureteral stricture caused by deep infiltrating endometriosis accompanied by severe kidney-induced arterial hypertension. In March 2022, the patient underwent both laparoscopic excision/evaporation of deep infiltrating endometriosis from the left ovarian fossa and left ureterolysis, followed by an ureterorenoscopic dilatation of the left ureter via the placement of an Allium self-expandable stent. **Results**: This stent was successfully removed 18 months later. A computed tomography check-up confirmed normal ureteral patency with no signs of endometriosis. Elevated blood pressure also resolved. **Conclusions**: Deep infiltrating endometriosis can lead to asymptomatic yet serious complications. A successful treatment of ureteral endometriosis may require multidisciplinary management, including a simultaneous laparoscopic and ureterorenoscopic approach. Ureteral stent placement is a minimally invasive state-of-the-art solution for ureteral stricture(s) and should be considered the first choice in women of reproductive age suffering from ureteral deep infiltrating endometriosis.

## 1. Introduction

Endometriosis is a medical condition that refers to the ectopic endometrium-like tissue found outside the uterus. It is responsible for chronic inflammation both at the site of the lesion’s presence and throughout the body. Estimates indicate that endometriosis affects 10% of the population of women of reproductive age [1].

Endometriosis can be asymptomatic, even in cases of significant anatomical progression. The urinary tract is involved in 1–5.5% of cases. Then, the urinary bladder is the most commonly affected organ, accounting for 70–85% of the cases, whereas the ureter is involved in 9–23% [2]. Ureteral endometriosis is most common in young patients aged 30–35 years. The disease frequently manifests unilaterally, with a predilection to affect the left-hand side of the body. The distal segment of the ureter is usually involved, mainly in the ovarian fossa, 3–4 cm above the ureterovesical junction. A treatment delay of ureteral endometriosis may result in hydronephrotic kidney damage and functional impairment [3].

Herein, we present a case of endometriosis silently infiltrating the ureter in a woman of reproductive age, causing ureteral stricture and kidney-induced arterial hypertension. We demonstrate a state-of-the-art minimally invasive treatment involving both the intraperitoneal and transvesical approach. We draw attention to a possible management of ureteral strictures caused by deep infiltrating endometriosis with a ureteral stent placement in order to regain normal urinary tract functioning and prevent serious systemic complications. The presentation was written in line with the SCARE 2020 guidelines for surgical case reports [4].

## 2. Case Study

**Figure 1:**
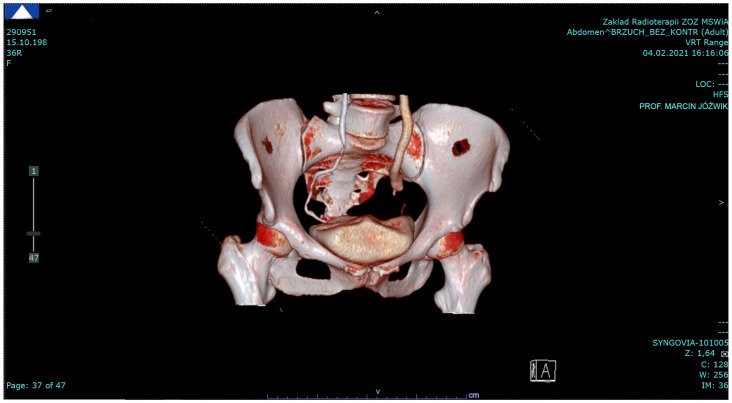
A preoperative CT scan demonstrating the normal right ureter and the left ureter dilated above its stricture at the ovarian fossa level (4 February 2021).

A 36-year-old female patient (G1, P1: Cesarean section in 2015) presented to the Gynecologic Oncology and Urogynecology Department in March 2021 for left-hand side lower abdominal pain. She had a history of mild left hydronephrosis in a 7-year observation complicated by recurrent urinary tract infections and mild hypothyroidism kept well under control with oral medications. The patient had undergone laparoscopic removal of a benign cyst in her left ovary in 2014. The pelvic examination and transvaginal ultrasound were normal. A computed tomography (CT) scan of the abdomen and pelvis revealed a mildly dilated kidney on the left side, and the left ureter distended to 10 mm in diameter over a stricture at the ovarian fossa level (Figure 1).

Based on the clinical picture and radiologic examinations, the patient was initially scheduled for endoscopic treatment. Yet, the surgical procedure planned for March 2021 had to be postponed due to the detection of an asymptomatic arterial hypertension reaching 190/110 mm Hg, and she was referred to a cardiologist for treatment. In January 2022, she was seen again with the normalized blood pressure values, confirmed with a Holter ambulatory blood pressure monitoring with a mean reading of 125/87 mm Hg. In March 2022, the patient underwent a combined endoscopic procedure: laparoscopic removal of deep infiltrating endometriosis of the left ovarian fossa, left ureterolysis, as well as ureterorenoscopic dilatation of the left ureter with an Allium 20-centimeter-long self-expandable stent (Allium LTD, Caesarea, Israel).

**Figure 2:**
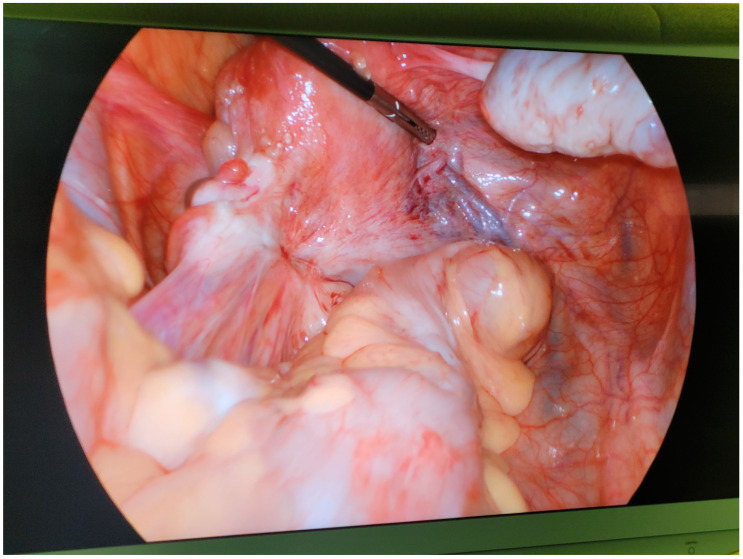
Endometriotic adhesions and fibrosis involving the left ovary and tube, sigmoid colon, and peritoneum found at laparoscopy (27 March 2022).

During the laparoscopic stage of the procedure, old postoperative adhesions in the area of the left ovary were found (Figure 2) and removed. Deep infiltrating endometriosis with associated fibrosis of the left ovarian fossa was confirmed, with the ureter trapped in a thick adhesion between the posterior uterine wall and sigmoid colon. The ureter was released, and the accompanying endometriosis was dissected by means of evaporation and cold resection to a degree that the ureter could regain its appropriate lumen. The walls of the left ureter and all adjacent blood vessels, particularly the left uterine artery, remained uninjured.

During the subsequent stage of the procedure, the ureterorenoscope was inserted through the urethra and urinary bladder to reach the left ureteral stenosis. The camera revealed a normal bladder mucosa and normal ureteral orifices. A soft-tip guidewire was placed into the left renal pelvis and a Charr 14 ureteral dilator inserted. The dilator found resistance located about 2 to 3 cm above the ureteral orifice to the bladder, which corresponded to the clinically confirmed stenosis near the posterior uterine wall. The ureter was dilated under X-ray control by advancing the dilator by approximately 20 cm. The dilator removed, an allium stent was inserted on the guidewire under X-ray control and placed so that an approximately 1 cm fragment of the stent remained in the bladder lumen. The guidewire delivery system was then removed. The control X-ray confirmed the proper position of the stent and the optimal dilatation of the site of the former ureteral obstruction. Throughout the procedure, the patient’s urine remained clear.

The surgical and perioperative course was uneventful. A control ultrasound confirmed full decompression of the left ureter and kidney. The patient was discharged on the second postoperative day in good condition.

**Figure 3:**
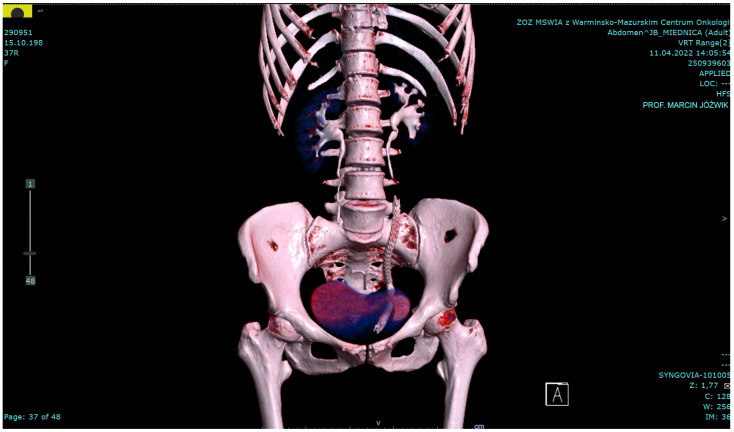
A postoperative CT scan showing the normal right ureter and a 20-centimeter-long allium stent in place in the left ureter and dilating it. Postoperatively, the stent migrated slightly into the urinary bladder, and its 5 cm end is visible in the lumen (11 April 2022).

At the follow-up visit in April 2022, the patient reported lower abdominal pain aggravated by sitting, walking, and micturition, as well as urethral discomfort. In a CT scan, a slight stent migration was noted with an approximately 5 cm fragment of the allium stent present in the bladder lumen (Figure 3).

**Figure 4:**
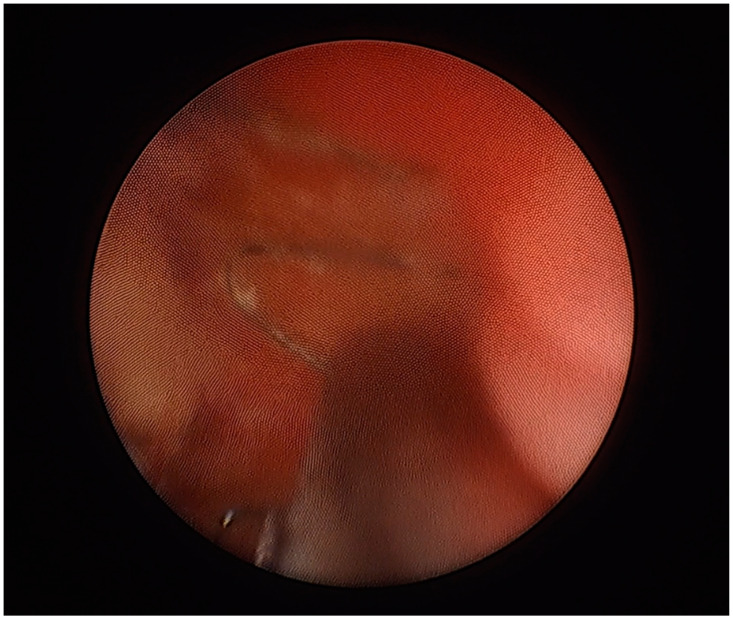
A cystoscopic view of the ureteral stent protruding into the urinary bladder lumen. The stent was trimmed using simultaneous cystoscopic and suprapubic intravesical access (13 May 2022).

A circa 3 cm protruding fragment of the stent (Figure 4) was removed from the bladder lumen by means of a cystoscopy combined with a suprapubic bladder endoscopy. The procedure fully resolved the complaints previously reported by the patient. Her blood pressure remained within normal limits.

**Figure 5:**
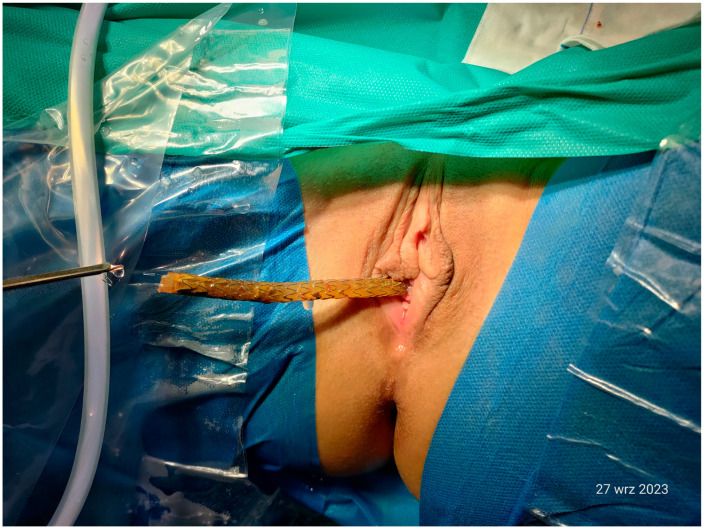
The transurethral removal of the calcified stent (27 September 2023).

**Figure 6:**
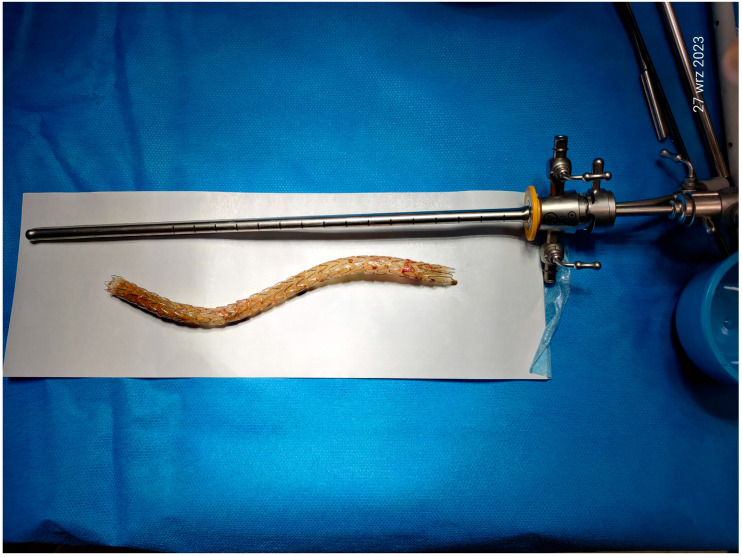
The removed device; note a trimmed end and calcified walls (27 September 2023).

First signs of stent calcification appeared in January 2023. The patient declared experiencing mild left kidney pains. Radiologic check-up excluded any signs of left ureter dilation, and she remained in observation. However, when seen in August 2023, she reported excretion of tiny ‘sand-like’ mineral sediments in her urine, and calcifications on the ureteral stent were confirmed in a following CT scan. Cystoscopic removal of the stent followed in September 2023. It was carried out with no complications. The removed prosthesis showed abundant calcifications (Figure 5 and Figure 6).

Another check-up followed in October 2023. The patient declared a mild discomfort (2 points out of 10 in the NRS, Numerical Rating Scale) in the left lumbar area, and showed a bacteria-positive urinary test. These resolved with oral furazidine treatment. A control CT scan demonstrated normal kidneys, no urinary retention, and normal left ureter with no concrements. The patient’s serum creatinine concentrations throughout the treatment and follow-up were all normal (Table 1). As of September 2024, she continues to be well.

## 3. Discussion

Urinary tract endometriosis is a rare complication. It can often be asymptomatic, or symptoms may mimic other conditions, thus making the prompt diagnosis more difficult [5,6].

Recurrent urinary tract infections have many contributing factors, endometriosis being one of them. In cases of unilateral ureteral obstruction in women of reproductive age, it is worthwhile to consider endometriosis in the differential diagnosis, once renal lithiasis has been excluded.

The early detection of ureteral endometriosis and the implementation of multidisciplinary gynecologic and urologic treatment not only prevent the further local progression of the disease, but also help in protecting other organs from endometriotic spread, the kidneys in particular. Internal complications such as medical treatment-resistant arterial hypertension can also be avoided [2,3].

To date, a typical surgical management of ureteral endometriosis has included ureterolysis, ureterotomy with end-to-end anastomosis, or ureteroneocystostomy. The ureterolysis alone for moderate to severe ureteral obstruction may be insufficient, resulting in the persistence or recurrence of symptoms, especially when the obstruction of the ureter prevails. Ureteroneocystostomy has a lower recurrence rate, but is associated with higher perioperative and postoperative complication rates [7], including anastomotic leak, ureteral fistula, and infection [8,9]. Reviews point out that hormonal therapies, such as gonadotropin-releasing hormone agonists and oral contraceptives, tend to be a rather temporary measure, yet have some role in a preoperative setting or if the patient is unsuitable for surgery, as well as in postoperative treatments [10].

In the presented case, we chose surgery as the first-line treatment due to the presence of hydronephrosis complicated by hypertension; this is where the ureteral obstruction seriously impacted the kidney’s function. We performed external ureterolysis with the implantation of a stent to the ureteral lumen to decompress the organ and prevent obstruction recurrence. Ureterorenoscopic dilatation and stent placement were integral components of the surgical procedure which successfully addressed the ureteral stenosis, leading to the resolution of both anatomical hydronephrosis and functional hypertension. A postoperative stent displacement into the bladder manifested in a lower abdominal pain. The complication was promptly recognized, and the stent’s fragment was effectively trimmed via cystoscopic access, resulting in the resolution of the patient’s symptoms. The allium stent has an intravesical anchor in its structure, yet the displacement of the device was observed in other studies. From a multicenter study in four countries, Moskovitz et al. noted its migration to the bladder in seven (14.3%) out of forty-nine placements [11]. In the Weinberger study, it was seen in four out of ten placements [12]. In other words, this situation requires awareness and vigilance.

A careful repeat search of electronic databases PubMed/Medline and Scopus in October 2024 for the search term ‘self-expanding ureteral stent for endometriosis’ found only one citation of a British study on the thermo-expandable nickel-titanium alloy stent Memokath 051 that was used in a singular case of extraluminal endometriosis [13]. In contrast, the allium stent is not only made of a particularly elastic nickel–titanium alloy, nitinol, but the entire device is covered with a biocompatible, biostable polymer to make it a nonpermeable tube to prevent tissue ingrowth into the lumen and early encrustation [11]. To date, it was applied for ureteric stenosis following surgery/radiation therapy for gynecologic malignancy, surgical and topical treatment for bladder cancer, ureteroenteric anasthomosis stricture after urinary diversion, endoscopic treatment of ureteral calculi, ureterocutaneostomy stricture, and renal transplantation [11,12]. A recent Chinese study added as indications congenital ureteropelvic junction obstruction, surgery/radiation therapy for rectal malignancy, retroperitoneal fibrosis, and trauma [14]. The stent was also used for ureteral strictures of unknown origin and due to aorto-bifemoral stent insertion [15]. Therefore, its use for deep infiltrating ureteral endometriosis seems to be a novel application, as is the dual endoscopic route of the surgery: laparoscopic excision of endometriosis and ureterolysis, followed by ureterorenoscopic dilation and the stent placement. In line with the SCARE guidelines [4], the strength of the presented novel surgical approach represents a truly minimally invasive technique, offering a viable alternative to more-invasive surgeries. The involvement of gynecologic and urologic expertise ensures a holistic approach to the condition. Yet, inherent to case studies, these findings may not be generalizable due to the single-patient focus, and additional studies are needed to establish broader clinical guidelines. Further exploration of minimally invasive techniques in similar cases of challenging presentations of endometriosis is encouraged.

## 4. Conclusions

Laparoscopic endometriosis excision and ureterolysis, as well as ureteral stent placement, require surgical proficiency in endoscopy to avoid injury to adjacent structures and achieve adequate decompression of the ureter. Such an approach seems to be an optimal solution for cases of endometriosis with ureteral stenosis. Close postoperative monitoring is essential to promptly identify and manage complications of this novel technique, such as stent-related issues, thus ensuring long-term recovery and patient satisfaction.

## Figures and Tables

**Table 1:** A chronological record of the patient’s blood creatinine concentrations.

Date	28 March 2021	11 January 2022	14 March 2022	9 April 2022	12 May 2022	17 January 2023	26 September 2023	14 October 2023
Serum creatinine (mg%)	0.79	0.57	0.68	0.74	0.75	0.72	0.83	0.85

## Data Availability

The data presented in this study are stored in the hospital registry.

## References

[1] Shafrir A.L., Farland L.V., Shah D.K., Harris H.R., Kvaskoff M., Zondervan K., Missmer S.A. (2018). Risk for and consequences of endometriosis: A critical epidemiologic review. Best Pract. Res. Clin. Obstet. Gynaecol..

[2] Berlanda N., Vercellini P., Carmignani L., Aimi G., Amicarelli F., Fedele L. (2009). Ureteral and vesical endometriosis. Two different clinical entities sharing the same pathogenesis. Obstet. Gynecol. Surv..

[3] Raimondo D., Mabrouk M., Zannoni L., Arena A., Zanello M., Benfenati A., Moro E., Paradisi R., Seracchioli R. (2018). Severe ureteral endometriosis: Frequency and risk factors. J. Obstet. Gynaecol..

[4] Agha R.A., Franchi T., Sohrabi C., Mathew G., Kerwan A. (2020). The SCARE 2020 guideline: Updating consensus Surgical CAse REport (SCARE) guidelines. Int. J. Surg..

[5] Uccella S., Cromi A., Casarin J., Bogani G., Pinelli C., Serati M., Ghezzi F. (2014). Laparoscopy for ureteral endometriosis: Surgical details, long-term follow-up, and fertility outcomes. Fertil. Steril..

[6] Ceccaroni M., Ceccarello M., Caleffi G., Clarizia R., Scarperi S., Pastorello M., Molinari A., Ruffo G., Cavalleri S. (2019). Total laparoscopic ureteroneocystostomy for ureteral endometriosis: A single-center experience of 160 consecutive patients. J. Minim. Invasive Gynecol..

[7] Barra F., Scala C., Biscaldi E., Vellone V.G., Ceccaroni M., Terrone C., Ferrero S. (2018). Ureteral endometriosis: A systematic review of epidemiology, pathogenesis, diagnosis, treatment, risk of malignant transformation and fertility. Hum. Reprod. Update.

[8] Chudzinski A., Collinet P., Flamand V., Rubod C. (2017). Ureterovesical reimplantation for ureteral deep infiltrating endometriosis: A retrospective study. J. Gynecol. Obstet. Hum. Reprod..

[9] Camanni M., Bonino L., Delpiano E.M., Berchialla P., Migliaretti G., Revelli A., Deltetto F. (2009). Laparoscopic conservative management of ureteral endometriosis: A survey of eighty patients submitted to ureterolysis. Reprod. Biol. Endocrinol..

[10] Nezhat C., Falik R., McKinney S., King L.P. (2017). Pathophysiology and management of urinary tract endometriosis. Nat. Rev. Urol..

[11] Moskovitz B., Halachmi S., Nativ O. (2012). A new self-expanding, large-caliber ureteral stent: Results of a multicenter experience. J. Endourol..

[12] Weinberger S., Hubatsch M., Klatte T., Neymeyer J., Friedersdorff F. (2023). The Allium ureteral stent for the treatment of ureteral complications following renal transplantation—A single-center, single-surgeon series. J. Clin. Med..

[13] Arya M., Mostafid H., Patel H.R., Kellett M.J., Philp T. (2001). The self-expanding metallic ureteric stent in the long-term management of benign ureteric strictures. BJU Int..

[14] Su B., Hu W., Xiao B., Liu Y., Zhang G., Tang Y., Li J. (2024). Long-term outcomes of Allium ureteral stent as a treatment for ureteral obstruction. Sci. Rep..

[15] Avitan O., Bahouth Z., Shprits S., Gorenberg M., Halachmi S. (2022). Allium ureteral stent as a treatment for ureteral stricture: Results and concerns. Urol. Int..

